# Magnetically Retrievable Nanoparticles with Tailored Surface Ligands for Investigating the Interaction and Removal of Water-Soluble PFASs in Natural Water Matrices

**DOI:** 10.3390/s25144353

**Published:** 2025-07-11

**Authors:** Yunfei Zhang, Jacqueline Ortiz, Shi He, Xianzhi Li, Bableen Kaur, Bing Cao, Zachariah Seiden, Shuo Wu, He Wei

**Affiliations:** 1 Department of Chemistry and Biochemistry, California State University Fresno, 2555 E San Ramon Ave, Fresno, CA 93740, USA; yunfei@mail.fresnostate.edu (Y.Z.); jacortiz@mail.fresnostate.edu (J.O.); xianzhi97@mail.fresnostate.edu (X.L.); kaurbableen609_@mail.fresnostate.edu (B.K.); 2 Department of Electrical and Computer Engineering, California State University Fresno, 2320 E San Ramon Ave, Fresno, CA 93740, USA; she3@mail.fresnostate.edu (S.H.); shuowu@mail.fresnostate.edu (S.W.); 3 Z18 Engineering, 1164 Winding Way Drive, Knoxville, TN 37923, USA; b.cao@z18engineering.com (B.C.); z.seiden@z18engineering.com (Z.S.)

**Keywords:** per- and polyfluoroalkyl substances (PFASs), perfluorooctanoic acid (PFOA), iron oxide magnetic nanoparticle (MNP), ligand-mediated magnetic separation, nanoparticle–PFAS interactions

## Abstract

Per- and polyfluoroalkyl substances (PFASs) are synthetic chemicals widely used in industrial applications and have become persistent environmental contaminants due to their chemical stability. Water-soluble PFASs with fewer than ten carbon atoms, such as perfluorooctanoic acid (PFOA), are particularly concerning because of their high solubility in water, environmental mobility, and resistance to degradation. In this work, we present an eco-friendly Fe_3_O_4_ magnetic nanoparticle (MNP)-based platform for the detection and removal of PFOA from water. The synthesized iron oxide MNPs exhibit rapid and strong magnetic responsiveness, enabling efficient magnetic separation for both PFOA detection and removal. To optimize surface affinity for PFOA, we functionalized the MNPs with distinctive ligands, including polyethylene glycol (PEG), β-cyclodextrin (βCD), and dopamine (DA). Among these, PEG and DA showed notable binding affinity toward PFOA, as confirmed by infrared spectroscopy and colorimetric assays. After incubation with the functionalized MNPs followed by magnetic retrieval, we achieved over 90% PFOA removal efficiencies, demonstrating the potential for future research in PFAS remediation technologies. Importantly, the system was validated using deionized, tap, and lake water, all of which yielded comparable and promising results. This study provides a promising, eco-friendly, and recyclable nanomaterial platform for investigating the crucial role of surface chemistry in nanoparticle–PFAS interactions through ligand-mediated magnetic separation.

## 1. Introduction

Per- and polyfluoroalkyl substances (PFASs) comprise a vast class of over 10,000 synthetic fluorinated compounds that have been widely utilized in industrial applications, such as firefighting foams, stain-resistant textiles, and non-stick cookware, due to their exceptional thermal stability, hydrophobicity, and chemical resistance [1,2]. However, these same properties that make PFASs industrially valuable also render them highly persistent in natural environments, earning them the moniker “forever chemicals.” Extensive research has revealed that even trace exposure to PFASs can lead to adverse health outcomes, including immune dysfunction, developmental toxicity, endocrine disruption, and increased risks of certain cancers [3]. Water-soluble PFASs, such as perfluorooctanoic acid (PFOA) or perfluorooctane sulfonic acid (PFOS), are now recognized as highly mobile in aquatic environments and just as chemically persistent, making them extremely difficult to remove or monitor effectively [1,4]. Their high water solubility exacerbates environmental spread and presents a significant analytical challenge in detecting them at low concentrations in complex matrices such as groundwater and even drinking water [5,6]. Therefore, the development of sensitive, selective, and field-deployable sensing strategies for water-soluble PFASs is imperative to ensure regulatory compliance and mitigate environmental and human health risks [7,8]. Current PFAS detection techniques are primarily dominated by laboratory-based methods, including liquid chromatography coupled with tandem mass spectrometry (LC-MS/MS), ion chromatography (IC), and high-resolution mass spectrometry (HRMS) [4,9]. While these methods are capable of detecting PFASs at parts-per-trillion (ppt) levels, they require expensive instrumentation, trained personnel, and rigorous sample pre-treatment, opening up opportunities for the development of alternative techniques [5,10]. In this regard, various alternative sensing technologies have been explored. Electrochemical sensors, for example, offer rapid response times and miniaturization potential but are often limited by electrode fouling and cross-reactivity in real samples [8,11]. Fluorescence-based sensors exhibit high sensitivity but can suffer from photobleaching, signal drift, and interference from organic matter or coexisting ions [9,12]. Surface-enhanced Raman scattering (SERS) platforms provide molecular fingerprint specificity but require complex surface modification and exhibit batch-to-batch variability [10,13]. Additionally, many sensing platforms struggle to effectively capture and concentrate water-soluble PFASs due to their weak hydrophobic interactions and low affinity for conventional recognition elements [14]. Consequently, there remains a pressing need for sensing systems that combine sensitivity, specificity, portability, and enrichment capability to achieve reliable detection of trace PFASs.

In response to these challenges, magnetic nanoparticles (MNPs) have emerged as a highly promising solution due to their unique ability to integrate separation, concentration, and signal transduction into a single platform [11,15]. Their intrinsic magnetic properties enable rapid and efficient separation from aqueous samples using an external magnetic field, thereby reducing background interference and simplifying sample processing [12]. Furthermore, MNPs can be easily functionalized with a wide range of ligands, polymers, and molecular receptors, enabling the design of highly selective binding interfaces tailored to specific PFAS structures [13,16]. This modular surface chemistry facilitates the construction of versatile sensing platforms, where MNPs serve not only as preconcentrates but also as recognition and signal transduction units in optical, electrochemical, or piezoelectric detection schemes. MNP-based platforms have demonstrated high enrichment efficiencies, often achieving orders-of-magnitude improvements in detection limits when compared to traditional methods, particularly for low-abundance analytes, such as water-soluble PFASs [17,18]. Moreover, the reusability and recyclability of MNPs make them well-suited for sustainable sensing applications, aligning with increasing demands for green and cost-effective analytical technologies [19]. These advantages position MNPs as an ideal candidate for developing field-deployable PFAS sensing systems that are both robust and scalable. In recent years, considerable progress has been made in engineering magnetic nanomaterials for PFAS detection, primarily through the use of functionalized eco-friendly Fe_3_O_4_ (magnetite) nanoparticles, silica–magnetite core–shell systems, and molecularly imprinted magnetic polymers [14,20]. These materials have been tailored with fluorophilic, zwitterionic, or ionic surfactant-like ligands to selectively bind PFAS molecules through hydrophobic, electrostatic, or hydrogen bonding interactions [21,22]. For example, Fe_3_O_4_ nanoparticles modified with polyethyleneimine and fluorinated alkyl chains have demonstrated enhanced affinity toward water-soluble PFASs due to increased fluorophilic attraction and favorable charge interactions [15,23]. Similarly, silica-coated magnetic nanoparticles (MNPs) embedded with molecular imprinting sites have demonstrated high selectivity and binding capacity for specific PFAS structures, enabling detection down to nanomolar or sub-nanomolar levels in environmental water [16,24]. Some platforms have also integrated MNPs with fluorometric or colorimetric readouts to facilitate rapid, on-site detection of PFASs [25]. Despite these advancements, a need remains to improve the selectivity for PFASs, many of which escape conventional detection methods due to their low molecular weight and reduced partitioning behavior [5,18]. Hence, strategies that can enhance surface binding through synergistic ligand design and nanoparticle engineering continue to be the focus of active research.

Among various magnetic materials, Fe_3_O_4_ nanoparticles offer several key advantages that make them particularly attractive for PFAS sensing applications. One of their most notable strengths lies in their functionalization flexibility—the surface of Fe_3_O_4_ can be readily modified with diverse ligands such as polyhydroxy compounds, fluorinated polymers, or zwitterionic chains, thereby enabling selective binding of PFASs through multiple interaction modes, including hydrogen bonding, hydrophobic interactions, and electrostatic attraction [19,20,26]. This adaptability is crucial for targeting water-soluble PFASs, which often lack sufficient hydrophobicity to be effectively adsorbed by traditional sorbents. Polyhydroxy ligands, in particular, offer the added benefit of forming hydrogen bond networks with the polar headgroups of PFAS molecules, thereby enhancing affinity and capture efficiency [27]. Dopamine-functionalized iron oxide nanoparticles offer strong surface binding through catechol groups and introduce amine functionalities that enhance electrostatic interactions with negatively charged PFAS molecules, improving capture efficiency [28]. Furthermore, Fe_3_O_4_ surfaces exhibit enhanced oxidative stability and colloidal dispersibility when coated with appropriate surface modifiers, which prevent aggregation and preserve nanoparticle performance under environmental conditions [21,29]. These coatings also mitigate potential cytotoxicity and environmental hazards, thus enabling the development of low-toxicity, recyclable sensing systems suitable for repeated use [21,30]. Collectively, these features make Fe_3_O_4_-based MNPs an ideal platform for next-generation PFAS sensors, offering enhanced preconcentration, selectivity, stability, and environmental compatibility.

In this work, we present a robust and recyclable sensing platform based on Fe_3_O_4_ MNPs functionalized with polyhydroxy or dopamine ligands, specifically designed for the targeted capture and detection of water-soluble PFOA. Our system capitalizes on the combined advantages of magnetic enrichment, selective surface chemistry, and high aqueous stability to overcome the persistent analytical challenges associated with detecting and quantifying PFOA in complex environmental matrices. Notably, the functionalized Fe_3_O_4_ nanoparticles exhibit excellent magnetic recoverability, enabling efficient and straightforward recycling with a retention efficiency exceeding 90% over multiple sensing cycles. This high recyclability not only supports sustainability but also significantly reduces operational costs, making the platform attractive for routine environmental monitoring. Furthermore, by leveraging Fourier-transform infrared (FTIR) spectroscopy, we achieve rapid detection as low as 125 µg/mL PFOA. This accessible and instrumentally straightforward method enables the real-time assessment of PFOA contamination in aqueous samples, eliminating the need for extensive sample preparation or expensive analytical infrastructure. Crucially, surface functionalization with polyethylene glycol (PEG) ligands markedly enhances the nanoparticle affinity toward PFOA, enabling a removal efficiency exceeding 90% even at concentrations as high as 10 µg/mL, which are 10,000 times higher than those typically encountered in regulated environments. This improved adsorption capacity remains consistent over three consecutive sensing cycles, demonstrating the system’s robustness and long-term applicability for high-performance PFAS remediation. While our current system is not yet optimized for ultra-trace quantification, it is intentionally designed to assess PFOA removal at µg/mL concentrations as a proof of concept for low-cost, field-deployable screening in high-concentration scenarios [31,32,33]. Future work will focus on extending detection capabilities to environmentally relevant levels (e.g., ng/L or ppt range) by integrating high-sensitivity analytical techniques such as LC-MS/MS.

To validate the compatibility of this platform in real environmental conditions, we also tested PFOA removal using tap water collected from our laboratory at California State University, Fresno, and surface water obtained from a natural lake. The results confirmed that the ligand-functionalized MNPs retained high adsorption efficiency and calibration accuracy even in the presence of naturally occurring ions and organic content. Together, these attributes establish our Fe_3_O_4_-based MNPs functionalized with tailored surface ligands as a platform for studying the interaction and removal of water-soluble PFASs in various water systems. The defining characteristics of the optimal surface ligands may guide the future design of alternative MNP platforms, enabling improved performance in the targeted removal of PFASs from real-world water matrices.

## 2. Materials and Methods

### 2.1. Materials

Synthesis of MNPs: The synthesis of Fe_3_O_4_ magnetite nanoparticles was adapted from previously reported methods. In a typical procedure, magnetite nanoparticles (MNPs) were prepared via the co-precipitation of Fe^2+^ and Fe^3+^ ions at a molar ratio of 1:2 using sodium hydroxide (NaOH) as the precipitating agent [34]. The iron precursor solution was prepared by dissolving 0.2 mol FeCl_3_·6H_2_O (54.05 g) and 0.1 mol FeCl_2_·4H_2_O (19.89 g) in deionized water, then adjusting the total volume to 100 mL. Separately, an 8.5 M NaOH solution was prepared by dissolving 0.8 mol NaOH (32.00 g) in 94 mL of deionized water under vigorous magnetic stirring. The iron precursor solution was added dropwise into the NaOH solution under continuous stirring at 500 rpm, maintaining a reaction temperature of 25 °C for 30 min.

The final reaction mixture had a volume of approximately 200 mL and a pH of ~13.2. To purify the synthesized nanoparticles and neutralize the high alkalinity, the suspension was separated into ten 50 mL centrifuge tubes and centrifuged at 4000 rpm for 10 min. After discarding the basic supernatant, the black solid in each tube was resuspended in 50 mL of deionized water, vortexed, and sonicated until fully redispersed. This was followed by centrifugation. This washing process was repeated three times until the supernatant reached a neutral pH, confirmed using pH paper. The neutralized solids were then washed with ethanol, centrifuged again, and the resulting paste-like material was collected, vacuum-dried, and ground into a fine black powder for storage and subsequent use.

Surface Functionalization of MNPs: For the PEG-coated nanoparticles (PEG-MNPs), 5 mg of Fe_3_O_4_ MNPs was dispersed in 5 mL of deionized (DI) water containing poly(ethylene glycol) (PEG, MW 8000, from Sigma-Aldrich, St. Louis, MO, USA) at a concentration of 10 mg/mL. The mixture was vortexed briefly and subjected to sonication for 10 min in a 40 kHz ultrasonic cleaner from VEVOR (Rancho Cucamonga, CA, USA) to promote uniform dispersion and enhance ligand attachment to the nanoparticle surface. It was then stirred vigorously overnight at room temperature to complete the functionalization. To prepare β-cyclodextrin-coated nanoparticles (βCD-MNPs), 5 mg of Fe_3_O_4_ MNPs was dispersed in 5 mL of DI water containing β-cyclodextrin at a concentration of 10 mg/mL (M.W. 1134.987, from Sigma-Aldrich, St. Louis, MO, USA). The same sonication procedure (40 kHz, 10 min) was applied, followed by overnight stirring. Dopamine-functionalized nanoparticles (DA-MNPs) were synthesized by dispersing 5 mg of Fe_3_O_4_ nanoparticles in 5 mL of 10 mg/mL aqueous solution of NaOH neutralized dopamine hydrochloride from Sigma-Aldrich (St. Louis, MO, USA), with the identical sonication step (40 kHz, 10 min) followed by overnight stirring. All surface-functionalized nanoparticles were centrifuged at 3000 rpm for 5 min and then resuspended in DI water three times to remove free ligands. The final purified nanoparticles were either resuspended in a minimal volume of deionized water for subsequent use in the PFOA adsorption experiments or dried under vacuum for storage.

### 2.2. Methods

Material Characterization: X-ray photoelectron spectroscopy (XPS) was performed using a Nexsa X-Ray Photoelectron Spectrometer from Thermo Fisher Scientific (Waltham, MA, USA), and all spectra were calibrated to the C 1s transition at 285.00 eV. X-ray diffraction (XRD) analyses were performed on a PANalytical X’Pert Pro diffractometer from Malvern Panalytical (Malvern, UK). Transmission Electron Microscopy (TEM) images were collected by a Talos F200C G2 Transmission Electron Microscope (Thermo Fisher Scientific, Waltham, MA, USA), operating at 200 kV, with the samples deposited on copper grids (Ted Pella, 01813 from Ted Pella, Inc, Redding, CA, USA). Infrared absorption was performed using a Nicolet Avatar FTIR spectrometer from Thermo Nicolet Corporation (Madison, WI, USA) and spectra were recorded in the 400–4000 cm^−1^ range to determine the surface composition of magnetic particles. A Bruker Vertex 70 FTIR infrared spectrometer was also used for this purpose.

Magnetic Retrieval of MNPs after surface coating: To evaluate the magnetic retrieval efficiency of each type of MNP by mass, 100 mg samples of dried PEG-MNPs, βCD-MNPs, and DA-MNPs were each suspended in 20 mL of deionized water in separate 20 mL glass vials. A neodymium magnet was placed against the outer wall of each vial, inducing the superparamagnetic particles to migrate and aggregate along the magnet-exposed side. Once separation was complete, the supernatant was carefully decanted while the magnet was kept in place to retain the MNPs along the vial wall. The vials, along with the magnetically retained MNPs, were then dried in an oven at 50 °C for 2 h. After cooling to room temperature in a desiccator, the total mass of each vial containing the recovered MNPs was measured using an analytical balance. The mass of retrieved MNPs was calculated by subtracting the pre-recorded mass of the empty dry vial. This procedure was repeated four times for each MNP type, and the recovery mass was used to assess the magnetic retrieval efficiency after each step The results of this multi-cycle retrieval experiment showed consistent magnetic recovery performance over four cycles for PEG-MNPs, βCD-MNPs, and DA-MNPs, as detailed in the data presented later in this manuscript.

Magnetic retrieval of coated MNPs after PFOA absorbance: Before magnetic retrieval, 5 mg of pre-washed surface-functionalized Fe_3_O_4_ MNPs was soaked in 5 mL of aqueous PFOA solutions at concentrations of 0, 125, 250, 500, and 1000 µg/mL and incubated for 1 h at room temperature (~25 °C) under gentle stirring at 400 rpm. The selected concentration range was designed to evaluate the dose-dependent adsorption behavior of the MNPs and to simulate scenarios involving elevated PFOA levels often encountered in contaminated industrial wastewater [31,32,33]. The pH of the solution was maintained near neutral (pH~7) to reflect typical environmental conditions and minimize pH-induced variations in adsorption efficiency. Following the adsorption process, a neodymium magnet was placed adjacent to the container wall, prompting the MNPs to migrate and aggregate at the side due to their superparamagnetic nature. After complete magnetic separation (less than 5 min), with the magnet held firmly against the side of the container to secure the MNPs along the wall, the supernatant was carefully decanted to avoid disturbing the retained nanoparticle pellet. The MNP pellet was then washed three times with deionized water, and a magnet was used to separate and eliminate any residual unabsorbed species. Each washing step involved gentle resuspension followed by magnetic separation. The final purified nanoparticles were either resuspended in a minimal volume of water for further analysis or dried under vacuum for storage. All steps were performed at room temperature with care taken to minimize material loss.

Quantification of PFOA removal by methylene blue (MB) colorimetric method: Three types of MNPs—PEG-MNPs, βCD-MNPs, and DA-MNPs—were evaluated individually for their PFOA removal efficiency. For each MNP type and each water matrix (deionized water, tap water, and lake water), a 7 mL glass vial was prepared containing 4 mg of the corresponding MNP. Separately, a 4 mL aliquot of a 10 µg/mL PFOA solution—prepared using the same water type—was added to the vial and stirred with a magnetic stir bar for 1 h at room temperature to allow adsorption. After mixing, an N52-grade neodymium magnet was applied to the outside of the vial to immobilize the MNPs against the wall. The supernatant PFOA solution was then carefully removed and transferred to a new vial containing 4 mg of the same MNP type. This process of stirring, magnetic separation, and solution transfer was repeated across three successive treatment steps using a fresh 4 mg of the same MNP type in each cycle. After the third adsorption step, the final supernatant, representing the residual PFOA solution after three treatments, was collected and methylene blue used for quantification. To each sample, 200 µL of 1% aqueous methylene blue (MB) solution was added, and the mixtures were shaken vigorously to ensure complete complexation between methylene blue and residual PFOA. Subsequently, 4 mL of chloroform was added to each vial, followed by vigorous shaking to extract the methylene blue–PFOA complex into the organic phase. After phase separation, the chloroform layer was carefully collected, and its absorbance at 650 nm was measured using a Cary 60 UV-Vis Spectrophotometer from Agilent (Santa Clara, CA, USA).

To establish a calibration curve, standard PFOA solutions at concentrations of 0, 1, 2, 3, 4, 5, 6, 7, 8, 9, and 10 µg/mL were prepared in each of the three matrices (DI water, tap water, and lake water). Each standard was subjected to the same methylene blue complexation and chloroform extraction procedure described above. Absorbance readings at 650 nm were plotted against the known PFOA concentrations to generate a linear calibration curve. This curve was then used to determine the concentration of residual PFOA in the test solutions after MNP treatment. These matrix-specific calibration curves were then used to determine the concentration of residual PFOA in each test sample, ensuring accurate quantification in all three water types. The PFOA removal efficiency was calculated using the following equation:

$$\mathrm{Removal}\;\mathrm{efficiency}(\%)=\frac{C_{o}-C_{f}}{C_{o}}\times 100\%$$

where C_o_ is the initial PFOA concentration (10 µg/mL) and C_f_ is the final concentration after treatment.

## 3. Results and Discussion

**Figure 1:**
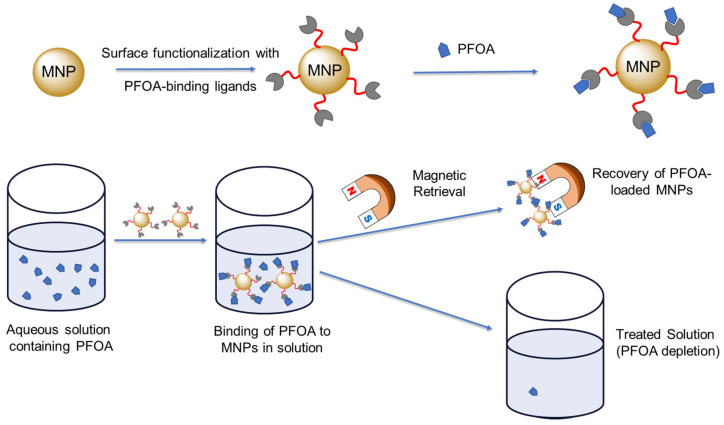
Schematic illustration of the functionalization of magnetic nanoparticles (MNPs) with surface ligands for PFOA binding and their magnetic retrieval process following PFOA removal.

Fe_3_O_4_ MNPs were synthesized via a co-precipitation method by adding NaOH to an aqueous mixture of Fe^2+^ and Fe^3+^ ions [34]. As shown in Figure 1, the synthesized MNPs were functionalized with three different ligand systems—poly(ethylene glycol) (PEG), β-cyclodextrin (βCD), or dopamine (DA)—to investigate their potential to enhance aqueous dispersibility and/or binding affinity toward water-soluble PFASs such as PFOA. PEG functionalization improved colloidal stability and reduced nonspecific interactions, while βCD coating introduced host–guest inclusion capability. A dopamine coating was achieved through strong coordination between catechol groups and iron oxide surfaces, providing a robust and hydrophilic interface. These three ligands were chosen because dopamine exhibits strong adhesion to iron oxide surfaces through its catechol groups and binds to PFASs through its amino groups. Moreover, PEG imparts colloidal stability and hydrophilicity, as well as affinity for PFASs through hydrogen bonding. Finally, the host–guest chemistry of βCD is expected to trap PFAS molecules within its cavity via hydrophobic interactions. Following functionalization, the modified MNPs were introduced into a PFOA-containing aqueous system, where ligand-mediated interactions facilitated the selective adsorption of PFOA.

**Figure 2:**
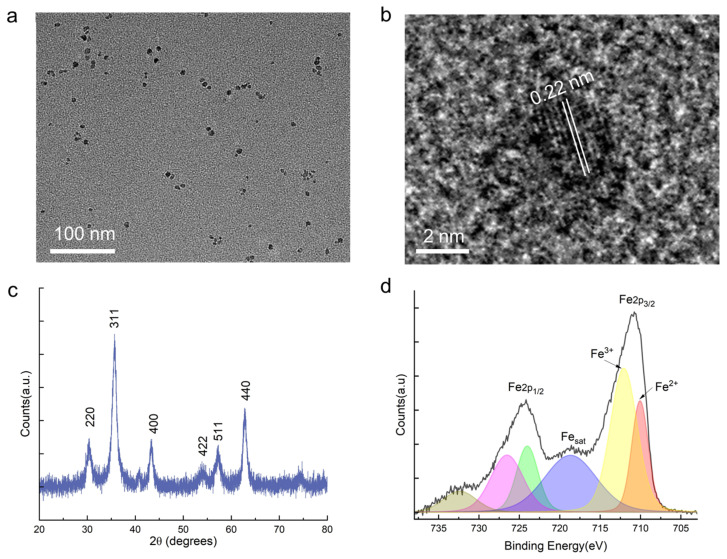
Structural and chemical characterization of Fe_3_O_4_ MNPs synthesized via co-precipitation. (**a**) Low-magnification TEM image showing well-dispersed Fe_3_O_4_ MNPs with minimal aggregation. (**b**) High-resolution TEM image displaying clear lattice fringes with an interplanar spacing of 0.22 nm, corresponding to the (311) plane of spinel Fe_3_O_4_. (**c**) XRD pattern and (**d**) XPS spectrum of the Fe 2p region of Fe_3_O_4_.

Synthesized Fe_3_O_4_ MNPs were comprehensively characterized to confirm their structural and chemical properties. As shown in the low-magnification transmission electron microscopy (TEM) image (Figure 2a), the 5 nm Fe_3_O_4_ MNPs are uniformly dispersed with minimal aggregation, exhibiting a relatively narrow size distribution centered below 10 nm. High-resolution TEM imaging (Figure 2b) further confirms the nanoparticle’s 5 nm inorganic diameter. It reveals clear lattice fringes with an interplanar spacing of 0.22 nm, which can be attributed to the (400) crystallographic plane of the spinel Fe_3_O_4_ phase, consistent with previous reports [35]. To assess the crystallinity and phase composition, X-ray diffraction (XRD) analysis was performed (Figure 2c). The diffraction pattern displays five distinct peaks located at 30.2°, 35.5°, 43.2°, 53.6°, 57.2°, and 62.6°, corresponding to the (220), (311), (400), (422), (511), and (440) planes, respectively [36,37]. These reflections match well with the standard cubic spinel structure of magnetite (Fe_3_O_4_) and agree with JCPDS No. 85–1436 [35,38]. The relative peak sharpness and intensity indicate the formation of highly crystalline nanoparticles, with no evidence of secondary phases such as maghemite or hematite. To probe the surface chemical states and oxidation environment of iron within the nanoparticles, X-ray photoelectron spectroscopy (XPS) was conducted (Figure 2d). The Fe 2p spectrum shows characteristic Fe 2p_3_/_2_ and Fe 2p_1_/_2_ peaks at binding energies of approximately 710.9 eV and 724.5 eV, respectively. Deconvolution of the Fe 2p_3_/_2_ peak reveals contributions from both Fe^2+^ and Fe^3+^ oxidation states, with the Fe^3+^ peak centered around 711.9 eV and the Fe^2+^ component appearing near 709.6 eV [34,35]. Additionally, the presence of a satellite feature (Fe_sat_) further supports the mixed-valent nature of Fe in the spinel structure. The coexistence of Fe^2+^ and Fe^3+^ is consistent with the stoichiometry of Fe_3_O_4_ and confirms the preservation of its electronic structure post-synthesis.

**Figure 3:**
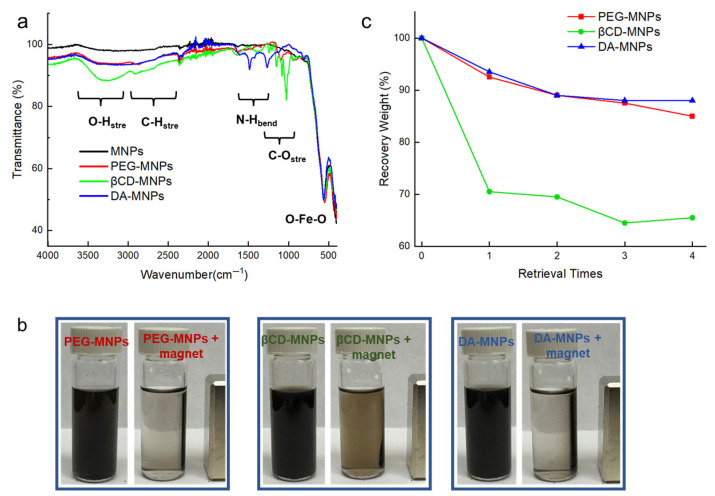
(**a**) FTIR spectra of uncoated and surface-functionalized Fe_3_O_4_ MNPs confirming successful coating with PEG, βCD, and DA. Characteristic stretching and bending vibrations corresponding to O–H, N–H, C–O, and Fe–O bonds are indicated. (**b**) Visual demonstration of magnetic separation ability for PEG-MNPs, βCD-MNPs, and DA-MNPs before and after exposure to an external magnetic field. (**c**) Recovery weight percentage of Fe_3_O_4_ MNPs after multiple retrieval cycles.

FTIR spectra of MNPs, PEG-MNPs, βCD-MNPs, and DA-MNPs, recorded in the range of 400–4000 cm^−1^, are presented in Figure 3a. All samples exhibit a distinct and intense absorption band below 600 cm^−1^, which corresponds to the stretching vibrations of Fe–O bonds in the spinel structure of magnetite, thus confirming the preservation of the core magnetic phase across all surface modifications [39]. A broad band spanning 3100–3600 cm^−1^ is evident for all samples and is primarily attributed to O–H stretching vibrations from adsorbed water, as supported by the water deformation band at ~1620 cm^−1^. Overlapping N–H stretching from ligand functionalities such as dopamine may also contribute to this region [40,41]. This band is particularly prominent in the DA-MNPs and βCD-MNPs, suggesting increased surface hydration or hydrogen bonding due to the polyhydroxy and amine-rich nature of dopamine and β-cyclodextrin, respectively. Additionally, a weaker shoulder at ~2900 cm^−1^, more clearly defined in PEG-MNPs, is attributed to the C–H stretching of methylene groups in the PEG backbone [42]. Characteristic peaks in the region of 1600–1450 cm^−1^ are assigned to N–H bending vibrations and water deformation modes, which are more intense in DA-MNPs, indicating the presence of surface-exposed amine functionalities [41]. Further absorptions observed between 1100 and 1000 cm^−1^ correspond to C–O stretching vibrations, particularly distinguishable in PEG-MNPs and βCD-MNPs, consistent with the ether and glycosidic linkages of PEG and βCD, respectively [42,43]. The coexistence of these distinct ligand-associated vibrational features, along with the retention of the Fe–O signature, confirms the successful functionalization of MNPs while preserving their core structure. The magnetic separation performance of surface-functionalized MNPs was visually assessed and is presented in Figure 3b. Three types of coated MNPs, PEG-MNPs, βCD-MNPs, and DA-MNPs, were individually dispersed in water, and an external magnet was applied to evaluate their separation behavior. In the absence of a magnet, all suspensions remained uniformly dark, indicating stable dispersion of MNPs. Upon exposure to an external magnetic field, however, a clear difference emerged. In the presence of a magnet, the PEG-MNPs (Figure 3b, left), βCD-MNPs (Figure 3b, middle), and DA-MNPs (Figure 3b, right) began to separate toward the magnet, leaving a visibly lighter supernatant behind. Notably, DA-MNPs and PEG-MNPs exhibited efficient clearance, resulting in a near-transparent treated solution. In comparison, βCD-MNPs still contained visible MNPs in the treated solution after magnetic retrieval. This strongly suggests that DA-MNPs and PEG-MNPs possess more robust magnetically responsive separation characteristics.

Quantitative analysis of magnetic retrievability across multiple cycles is shown in Figure 3c. The recovery weight percentages of PEG-MNPs, βCD-MNPs, and DA-MNPs were measured after repeated PFAS removal and magnetic retrieval cycles. As depicted, DA-MNPs maintained a recovery rate above 90% even after four retrievals, showing minimal loss of magnetic material. PEG-MNPs also demonstrated relatively high retrievability, though a slight decline was observed with increasing cycle number. In contrast, βCD-MNPs experienced a rapid decrease in recovery weight after the first cycle, stabilizing at approximately 68% in subsequent cycles. This discrepancy is attributed to variations in surface coating stability and magnetic responsiveness, which influence the agglomeration and sedimentation behavior under magnetic fields. These findings confirm that among the tested coatings, dopamine-functionalized MNPs offer the most robust recyclability and separation efficiency, making them highly suitable for repeated PFAS removal applications.

**Figure 4:**
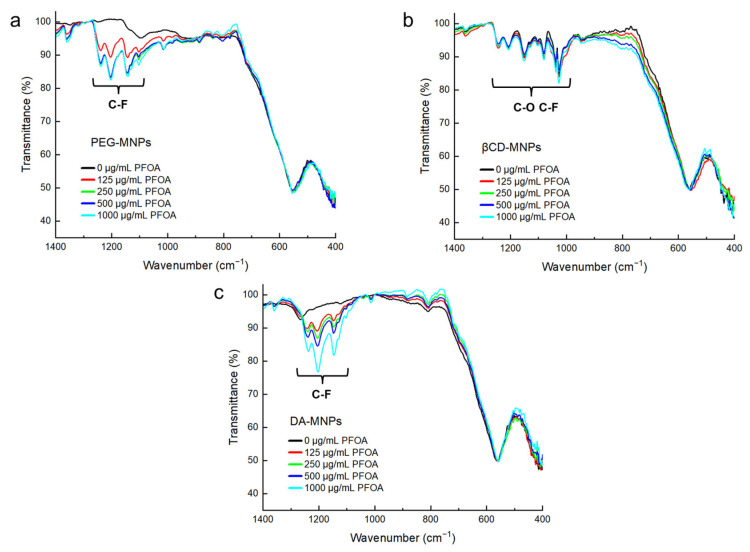
FTIR spectra of (**a**) PEG-MNPs, (**b**) βCD-MNPs, and (**c**) DA-MNPs before and after exposure to increasing concentrations of perfluorooctanoic acid (PFOA) (0–1000 µg/mL).

In the case of PEG-MNPs (Figure 4a), progressive increases in the intensity of absorption bands in the 1150–1250 cm^−1^ region were observed with increasing PFOA concentrations. These bands are attributed to the stretching vibrations of C–F bonds from the perfluorinated carbon chains of PFOA molecules [44,45]. The apparent correlation between PFOA concentration and C–F peak intensity indicates dose-dependent adsorption behavior on the PEG-functionalized surface. This is likely facilitated by van der Waals interactions and dipole–dipole attractions between the ether groups of PEG and the polar fluorinated moieties of PFOA. βCD-MNPs (Figure 4b), on the other hand, exhibited minimal spectral changes upon PFOA exposure. Although βCD possesses a hydrophobic cavity that can theoretically accommodate small organic molecules, the low signal increase in the C–F region suggests poor complexation efficiency with PFOA. This may be due to the relatively large molecular size and rigidity of PFOA compared to the dimensions of the βCD cavity, or insufficient molecular affinity to overcome steric and hydration barriers. Additionally, the C–O stretching vibrations around 1030–1150 cm^−1^, attributable to glycosidic linkages in βCD, showed negligible shifts, further indicating minimal interaction or structural perturbation upon PFOA contact [43,45]. Some spectral changes were observed for DA-MNPs (Figure 4c), which displayed substantial and concentration-dependent intensification of the C–F stretching region upon PFOA adsorption. Dopamine moieties, rich in catechol and amine functionalities, are known to form strong electrostatic interactions and hydrogen bonds with acidic or electronegative groups such as the carboxylate and fluorinated tail of PFOA. These interactions facilitate enhanced surface binding and molecular entrapment, resulting in a robust spectral signature in the fluorine-associated region. Furthermore, the higher surface polarity and amine density in DA-MNPs may contribute to improved surface wettability and accessibility for PFOA molecules, enhancing capture efficiency. Collectively, the FTIR results provide direct spectroscopic evidence of surface-specific interactions between PFOA and functionalized Fe_3_O_4_ nanoparticles. DA-MNPs exhibit the strongest and most consistent signals of PFOA uptake, followed by PEG-MNPs with moderate adsorption capability. βCD-MNPs demonstrate limited spectral response, suggesting lower PFOA capture efficiency under the tested conditions. These findings align well with magnetic separation and recovery data, reinforcing the excellent utility of DA-coated MNPs for efficient and reusable PFAS removal applications.

**Figure 5:**
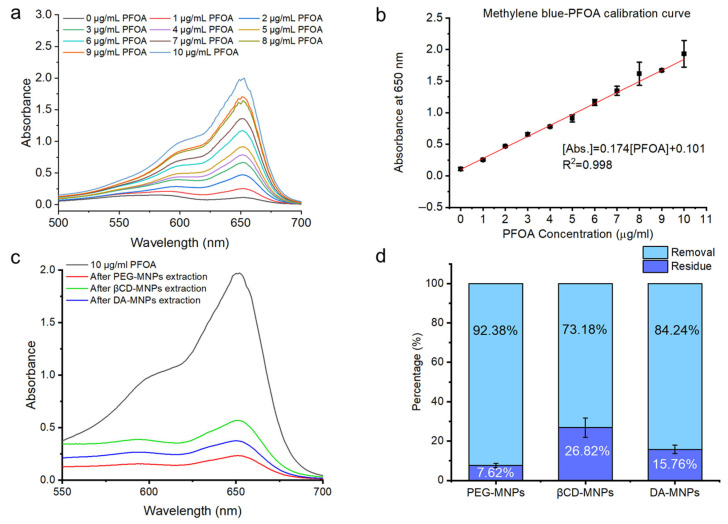
Quantitative comparison of PFOA removal efficiency using surface-functionalized Fe_3_O_4_ magnetic nanoparticles (MNPs): PEG-MNPs, βCD-MNPs, and DA-MNPs. (**a**) UV-Vis absorbance spectra showing increasing absorbance at ~650 nm with rising concentrations of PFOA (0–10 µg/mL). (**b**) Calibration curve for methylene blue–PFOA complex measured at 650 nm. (**c**) UV-Vis absorbance spectra of 10 µg/mL PFOA before and after treatment with three different magnetic nanoparticles: PEG-MNPs, βCD-MNPs, and DA-MNPs. (**d**) Bar chart representing the percentage of PFOA removed (light blue) versus remaining residue (dark blue) after treatment with each type of MNP. DA-MNPs.

To understand the effect of surface functionalization on the adsorption performance of MNPs toward PFOA, three types of surface-modified Fe_3_O_4_ MNPs—PEG-MNPs, βCD-MNPs, and DA-MNPs—were evaluated for their removal efficiency. In Figure 5, each experiment was conducted by adding 4 mg of freshly prepared MNPs to 4 mL of a 10 µg/mL PFOA aqueous solution. After a 1 h incubation at room temperature with gentle stirring (400 rpm), the mixture underwent adsorption and magnetic separation steps three times. The same procedure was sequentially repeated for three fresh MNP aliquots to simulate cumulative adsorption using a total of 12 mg of MNPs per formulation. The residual concentration of PFOA in the supernatant was determined using a validated methylene blue (MB) colorimetric assay [44]. In this method, PFOA at increasing concentrations (0–10 µg/mL) forms a stable ion pair with the cationic dye methylene blue (MB), which is extractable into chloroform and exhibits increasing absorbance at ~650 nm as measured by UV–Vis spectrophotometry, indicating successful complexation with MB, as shown in Figure 5a. Next, a calibration curve was established in the range of 0–10 µg/mL, yielding a linear response (R^2^ > 0.998), which ensured accurate quantification of PFAS removal within the tested concentration range (Figure 5b).

Among the tested nanomaterials, PEG-MNPs achieved the highest removal efficiency, exceeding 90% under the given conditions. DA-MNPs exhibited slightly lower performance, reaching approximately 84%, while βCD-MNPs showed the weakest efficiency at around 73%. Based on these values, the estimated adsorption capacities were 3.1 µg/mg for PEG-MNPs, 2.8 µg/mg for DA-MNPs, and 2.4 µg/mg for βCD-MNPs. This satisfactory performance of PEG-MNPs is attributed to the amphiphilic nature of polyethylene glycol, which promotes strong van der Waals and dipole–dipole interactions with both the hydrophobic perfluorinated tail and the polar carboxyl head group of PFOA. DA-MNPs also demonstrated high removal capacity, achieving approximately 85% uptake. The presence of catechol and amine groups on the dopamine-functionalized surface is believed to enhance adsorption through hydrogen bonding, electrostatic interaction, and surface complexation with the carboxylate group of PFOA. In contrast, βCD-MNPs exhibited significantly lower adsorption performance, with only ~70% removal efficiency and more than 25% of PFOA remaining in solution. Despite the known inclusion capabilities of β-cyclodextrin for various hydrophobic organic molecules, the rigid structure and relatively large molecular size of PFOA likely limit its encapsulation within the βCD cavity. Additionally, the absence of strong ionic or hydrogen bonding sites on the βCD surface may further hinder its interaction with PFAS species. Collectively, these results underscore the crucial role of surface chemistry and functional group accessibility in PFAS capture.

These differences in removal efficiency can be attributed to the distinct surface chemistries and molecular interaction mechanisms of the ligands. While PEG-MNPs provide highly efficient removal through amphiphilic interaction, and DA-MNPs offer multi-modal binding mechanisms, βCD-MNPs appear less suited for direct PFOA sequestration. The findings suggest that rational design of surface functionalities is essential to optimize MNP-based adsorbents for effective and selective PFAS remediation.

To assess the applicability and environmental compatibility of our assay platform, we collected two real-world water samples and evaluated PFOA removal performance under non-ideal conditions. Tap water was sampled from the laboratory at California State University, Fresno, and surface water was collected from a natural lake. These two matrices were selected to represent typical treated municipal water and surface freshwater, which contain organic matter and ions. Each sample was spiked with 10 µg/mL PFOA, and separate calibration curves were constructed using the same water matrices to account for potential matrix effects on the methylene blue–PFOA complexation assay.

**Figure 6:**
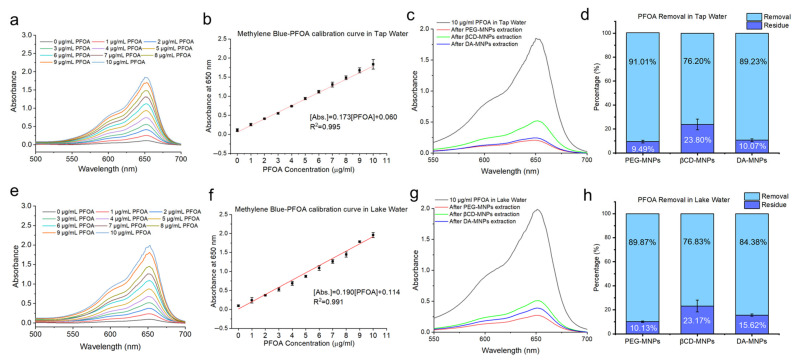
Quantitative comparison of PFOA removal efficiency using surface-functionalized Fe_3_O_4_ magnetic nanoparticles (MNPs) in real water matrices: tap water and lake water. (**a**) UV-Vis absorbance spectra showing increasing absorbance at ~650 nm with rising concentrations of PFOA (0–10 µg/mL) in tap water. (**b**) Calibration curve for methylene blue–PFOA complex measured at 650 nm in tap water. (**c**) UV-Vis absorbance spectra of 10 µg/mL PFOA in tap water before and after treatment with PEG-MNPs, βCD-MNPs, and DA-MNPs. (**d**) Bar chart representing the percentage of PFOA removed (light blue) versus remaining residue (dark blue) after treatment in tap water with each type of MNP. (**e**) UV-vis absorbance spectra showing increasing absorbance at ~650 nm with rising concentrations of PFOA (0–10 µg/mL) in lake water. (**f**) Calibration curve for methylene blue–PFOA complex measured at 650 nm in lake water. (**g**) UV-Vis absorbance spectra of 10 µg/mL PFOA in lake water before and after treatment with PEG-MNPs, βCD-MNPs, and DA-MNPs. (**h**) Bar chart representing the percentage of PFOA removed (light blue) versus remaining residue (dark blue) after treatment in lake water with each type of MNP.

As shown in Figure 6, UV-Vis calibration confirmed strong linearity in both systems (R^2^ = 0.995 for tap water, R^2^ = 0.991 for lake water). After treatment with functionalized MNPs, PEG-MNPs achieved the highest PFOA removal efficiencies—91.01% in tap water and 89.87% in lake water. DA-MNPs also demonstrated strong performance, achieving 89.23% and 84.38% removal, respectively. βCD-MNPs exhibited moderate but consistent removal with 76.20% in tap water and 76.83% in lake water. The corresponding adsorption capacities were 3.03 µg/mg (PEG-MNPs), 2.97 µg/mg (DA-MNPs), and 2.54 µg/mg (βCD-MNPs) in tap water; and 3.00 µg/mg, 2.81 µg/mg, and 2.56 µg/mg, respectively, in lake water. These results demonstrate that the assay maintains high adsorption performance even in the presence of background ions and organics. The strong correlation between calibration and removal performance supports the system’s potential as a robust, low-cost, field-adaptable assay for preliminary PFAS screening in environmental water systems.

The robust PFOA removal efficiency observed for PEG-MNPs compared to DA-MNPs and β-cyclodextrin-MNPs can be attributed to their distinct surface chemistries that dictate molecular-level interactions with PFOA. PEG chains possess amphiphilic characteristics due to the presence of both hydrophilic ether groups and hydrophobic alkyl backbones, which facilitate strong dipole–dipole interactions and van der Waals forces with both the carboxylic headgroup and perfluorinated tail of PFOA [15,26]. These interactions are enhanced by the high conformational flexibility of PEG chains, which enables better molecular accommodation and dynamic adaptation to the PFOA structure, resulting in surface crowding and cooperative adsorption effects [11,19]. Furthermore, the abundant C–O ether linkages in PEG serve as polar interaction sites for the electron-rich fluorinated moieties, effectively increasing binding affinity [18]. FTIR data from this study support this hypothesis, showing concentration-dependent intensification of C–F stretching bands for PEG-MNPs, indicating substantial molecular loading. The hydrophilic corona formed by PEG also aids in stabilizing MNP dispersion in aqueous environments, ensuring maximal surface exposure and minimizing aggregation, a critical factor for optimizing interaction kinetics and capacity [20,21]. In contrast, the moderate performance of DA-MNPs, which achieved approximately 85% removal efficiency, is driven by specific hydrogen bonding and electrostatic interactions between PFOA’s carboxyl group and the catecholamine functionalities of dopamine [22,27]. The catechol moieties contribute to π-π stacking and ion–dipole interactions that enhance surface binding; however, these interactions are more localized and less conformationally adaptive compared to PEG, which may limit molecular accommodation at high PFOA concentrations. Moreover, the higher surface polarity of DA-MNPs, while advantageous for hydrophilic headgroup recognition, may create repulsion against the hydrophobic fluorinated tail, reducing overall adsorption efficiency. βCD-MNPs displayed the weakest performance (~70%) due to steric mismatch and limited hydrophobic cavity dimensions that poorly accommodate the bulky, linear PFOA molecules [8,24]. Although βCD is known for its host–guest chemistry, the rigidity of its toroidal structure and the lack of cooperative binding sites for the acidic headgroups or fluorinated chains significantly impair complexation efficacy [14,17]. The negligible spectral shift in FTIR and reduced recyclability further support the limited molecular-level engagement. Collectively, these results underscore that PEG-MNPs outperform other ligand systems by leveraging synergistic amphiphilic interactions, a flexible surface architecture, and high colloidal stability, thereby establishing them as the most effective nanoplatform for PFOA sequestration under aqueous conditions.

## 4. Conclusions

In this study, we developed and systematically evaluated a series of surface-functionalized Fe_3_O_4_ magnetic nanoparticles (MNPs) for the targeted removal of water-soluble per- and polyfluoroalkyl substances (PFASs), specifically, perfluorooctanoic acid (PFOA). Our work focused on elevated PFAS levels commonly found in industrial waste streams to assess material capacity, surface interactions, and recyclability in simplified systems. Among the three ligand systems investigated—polyethylene glycol (PEG), dopamine (DA), and β-cyclodextrin (βCD)—functionalized MNPs exhibited superior removal efficiency, achieving over 90% PFOA extraction from aqueous solutions. This performance is attributed to the amphiphilic and conformationally flexible nature of PEG, which facilitates strong van der Waals, dipole–dipole, and hydrogen bonding interactions with both hydrophobic and polar regions of the PFOA molecule. DA-MNPs showed commendable binding via electrostatic and hydrogen bonding interactions. At the same time, βCD-MNPs demonstrated limited performance likely due to steric mismatch and poor host–guest compatibility with the PFOA structure. Beyond removal efficiency, all three nanoparticle types exhibited excellent magnetic retrievability, with PEG-MNPs and DA-MNPs retaining structural integrity over multiple usage cycles. Our findings highlight the crucial role of surface chemistry in determining nanoparticle–PFAS interactions and establish PEG-functionalized Fe_3_O_4_ MNPs as a recyclable material for PFAS remediation. Importantly, we validated the system’s robustness using real-world water matrices, which yielded comparable removal efficiencies to those in deionized water. This work not only offers mechanistic insight into molecular-level binding phenomena but also provides an alternative solution to address the growing environmental and public health challenges posed by PFAS contamination. While this study focuses on the removal performance of PFOA-rich effluents commonly generated by industrial processes, the current system provides an accessible, low-cost foundation for further development. Future directions will focus on expanding the ligand library to enhance PFAS interaction and specificity, as well as on integrating real-time sensing capabilities and LC-MS/MS-based quantification for trace-level, field-deployable applications.

## Data Availability

Data is contained within the article.
